# Applied physiology at the bedside: using invasive blood pressure as a true monitoring tool

**DOI:** 10.1186/s13613-025-01608-y

**Published:** 2025-12-10

**Authors:** Maxime Bertrand, Antoine Goury, Denis Chemla, Jean-Louis Teboul, Olfa Hamzaoui

**Affiliations:** 1 Unité de Médecine Intensive Et Réanimation Polyvalente, University Hospitals of Reims, 51100 Reims, France; 2 University of Reims Champagne-Ardenne, Unité PPF “Pharmacologie et Pathologies Fragilisantes” – UR 3801, 51100 Reims, France; 3 INSERM UMRS 999, Hôpital Marie Lannelongue, 133 Avenue de La Résistance, 92350 Le Plessis-Robinson, France; 4 University of Paris-Saclay, Le Kremlin-Bicêtre, France

**Keywords:** Blood pressure waveform, Diastolic arterial pressure, Systolic arterial pressure, Mean arterial pressure, Pulse pressure, Pulse pressure variation

## Abstract

Invasive arterial blood pressure (BP) monitoring is a cornerstone of hemodynamic assessment in critically ill patients. This review explores how the individual components of BP—systolic arterial (SAP), diastolic arterial (DAP), mean arterial (MAP), and pulse pressure (PP)—offer valuable insights into cardiovascular physiology and can be leveraged as real-time therapeutic tools in intensive care settings. A strong emphasis is placed on the technical requirements for accurate BP waveform interpretation and the physiological meaning of each BP component. PP is examined as a surrogate for stroke volume and a dynamic marker of fluid responsiveness, particularly in mechanically ventilated patients. DAP is discussed as a reflection of vasomotor tone, with clinical implications for guiding the initiation of vasopressors. The concept of diastolic shock index (DSI) and the newly proposed VNERi ratio (DAP/[Heart rate × norepinephrine dose]) are introduced as potentially superior markers for assessing vascular tone and vasopressor responsiveness, respectively. These indices may facilitate earlier identification of patients requiring escalation of vasopressor therapy, including the initiation of vasopressin in addition to norepinephrine. The review advocates for a physiology-driven, individualized approach to hemodynamic management, using invasive BP not merely as a safety parameter but as an actionable guide for precision resuscitation.

## Introduction

Invasive blood pressure (BP) is a readily measurable hemodynamic variable, akin to heart rate (HR) and oxygen saturation (SpO₂), and serves as a fundamental tool for bedside hemodynamic monitoring. The components of BP—systolic arterial pressure (SAP), diastolic arterial pressure (DAP), mean arterial pressure (MAP), and pulse pressure (PP, defined as the difference between SAP and DAP)—offer critical insights for both diagnostic evaluation and therapeutic decision-making.

This review highlights how these parameters can be leveraged as dynamic monitoring tools in intensive care settings to enable real-time therapeutic adjustments in therapy. Their continuous monitoring through an arterial catheter provides vital information for managing patients in the intensive care unit (ICU), ensuring optimal organ perfusion and tailoring interventions according to of individualized patients’ needs.

## Prerequisites for accurate interpretation

Before interpreting invasive BP values displayed on a monitor, it is imperative to validate the reliability of the measurements by assessing the quality of the BP waveform. Accurate waveform analysis is essential for ensuring the data’s integrity and its utility in evaluating the patient’s hemodynamic status. Distortions in the waveform, such as underdamping or overdamping, can lead to erroneous measurements and potentially inappropriate clinical decisions.

The transducer should have an adequate frequency response (natural frequency > 100 Hz), be correctly zeroed, and display no baseline drift.

### Characteristics of a high-quality blood pressure waveform

**Fig. 1:**
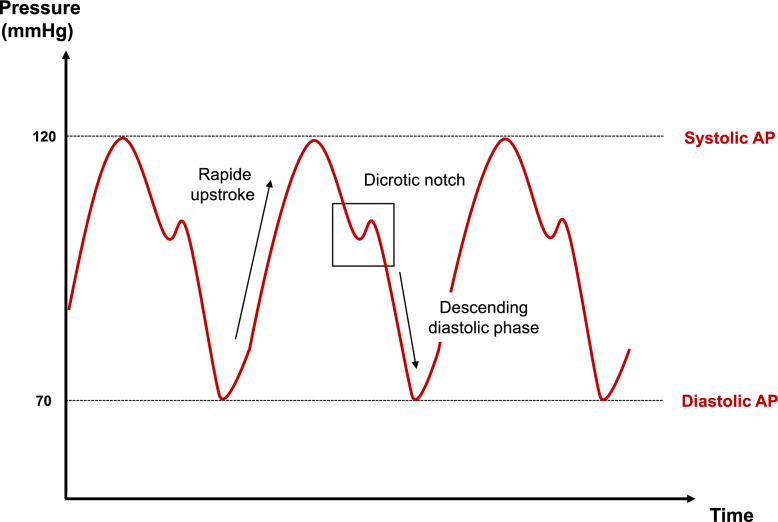
Normal blood pressure curve

A reliable BP waveform should exhibit specific characteristics (Fig. 1):

- *Rapid Upstroke*. Note that a slow upstroke should prompt the clinician to first rule out recording artifacts responsible for energy dissipation, before attributing to severe cardiocirculatory failure.
- *Dicrotic Notch.* It confirms proper aortic valve function and the absence of significant aortic regurgitation, which impacts on peripheral BP interpretation.
- *Descending Diastolic Phase*. The waveform's downward slope after the dicrotic notch should gradually approach the DAP without irregular oscillations. A smooth descent signals an appropriate return to baseline and excludes artifacts or interference.

By ensuring the integrity of the BP waveform, clinicians can rely on the data to guide precise therapeutic interventions and improve outcomes in ICU patients.

### Common artifacts and their correction

**Fig. 2:**
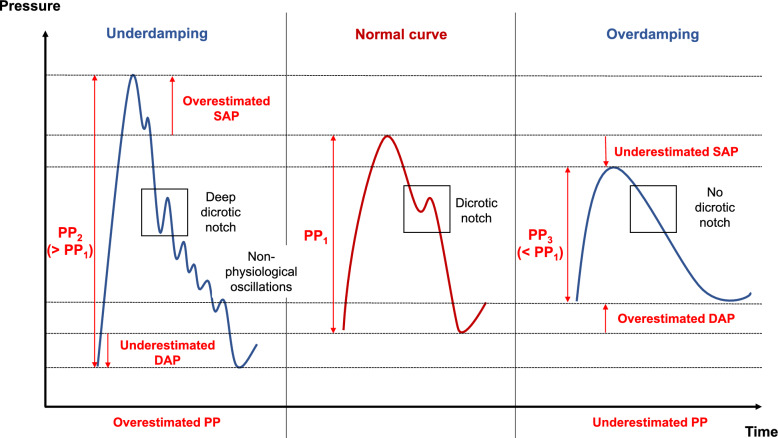
Blood pressure curve and damping artefacts

The accuracy of invasive BP measurements relies on the performance of the measurement system, which is influenced by factors such as the fluid dynamics of the system, the elasticity of the tubing, and friction. These factors determine the system’s natural frequency (pulse oscillations) and damping coefficient (oscillation decay) [1–3]. Variations in these parameters can lead to specific waveform artifacts, as outlined (Fig. 2):

- *Underdamping:* Multiple oscillations following the dicrotic notch, lead to an overestimation of the SAP (narrower and exaggerated peak of the systolic part of the BP curve), an underestimation of the DAP, and an overestimation of the PP.
- *Overdamping:* The waveform is excessively flattened, resulting in an underestimation of the SAP and an overestimation of the DAP.

### Corrective actions

**Fig. 3:**
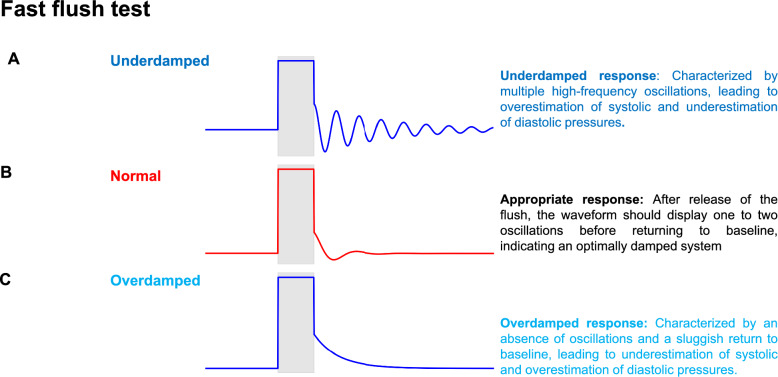
Fast flush test: the fast flush test consists of briefly activating the flush device (continuous pressure source of 300 mmHg) to produce a square wave followed by oscillations in the pressure tracing. The test allows assessment of the natural frequency and damping coefficient of the monitoring system. **A **Underdamped response; **B** Appropriate response; **C **Overdamped response

First, as damping errors can substantially alter interpretation of the blood pressure components, the fast-flush test should be performed systematically to validate the quality of the recording before clinical use (Fig. 3).

Other interventions can mitigate—and overdamping artifacts to ensure accurate measurements:

- *Addressing underdamping:*
  - Use short, rigid, and non-compressible tubing to minimize system elasticity.
  - Reduce the number of stopcocks and avoid unnecessary alterations to the transducer kit.
  - Stabilize the catheter to limit movement and consider incorporating adjustable damping devices to increase the damping coefficient when required.
- Resolving overdamping:
  - Identify and address common causes such as air bubbles, blood clots, or kinks in the catheter.
  - Reposition the patient’s wrist to optimize the catheter alignment.
  - Remove air bubbles or clots and replace the catheter or arterial site if the waveform remains unreliable.

### Site of measurement of invasive arterial blood pressure:

The MAP measured in the femoral artery was reported to be more often higher than the MAP measured in the radial artery in ICU patients including those with septic shock under high dose vasopressors [4]. The clinical impact of these differences (3 mmHg on average), is still unclear, suggesting no immediate need to alter current preferences for peripheral cannulation [4].

## Physiological insights into arterial blood pressure

### Aortic pressure

**Fig. 4:**
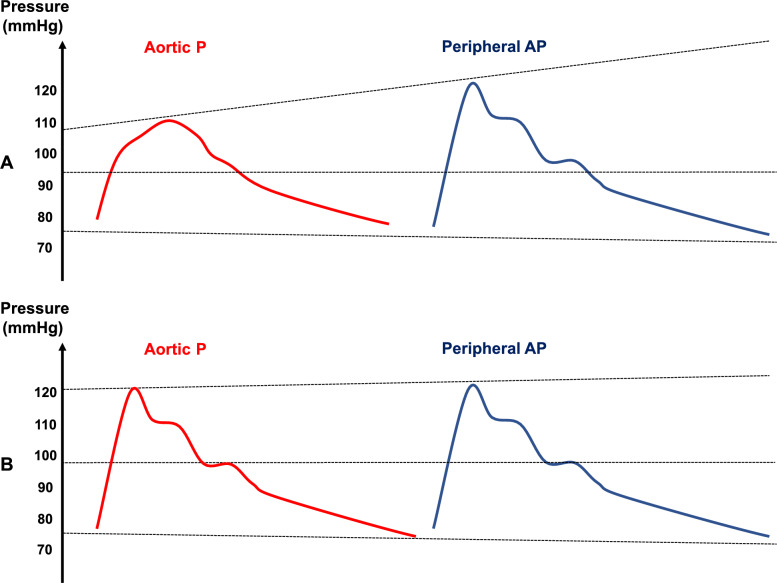
Pulse wave Amplification between aortic blood pressure and peripheral blood pressure: **A** In a young subject. **B** In an old subject

Arterial BP comprises two primary components: a steady component and a pulsatile component. The steady, or continuous, component is quantified by the MAP, which remains constant or minimally decreases from the aorta to the peripheral large arteries. This is due to the aorta and large arteries being elastic structures with high calibre, which results in low resistive function. The MAP depends essentially on the interplay between cardiac output (CO) and peripheral vascular resistance. The pulsatile component (PP) may increase from the aorta to the periphery due to the phenomenon known as PP amplification (PPA = peripheral PP/aortic PP) or pulse wave amplification (PWA) (Fig. 4), which will be described later.

At the population level, while peripheral BP provides essential information, evidence suggests that elevated aortic BP may be more closely associated with target organ damage and potentially with cardiovascular risk. Accordingly, the effects of antihypertensive treatment may be more accurately assessed by considering their central rather than peripheral actions. These issues, however, remain actively debated.

### Arterial compliance

**Fig. 5:**
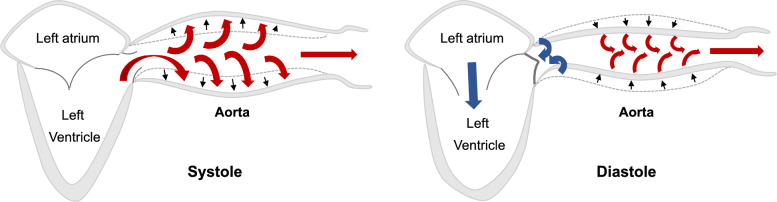
Physiological phenomenon that transforms discontinuous pulsed blood flow into continuous flow (Windkessel effect)

The aorta and large arteries are not passive conduits of resistance but dynamic elastic structures. During systole, a fraction of the stroke volume (SV) is directed peripherally (“systolic run-off”), while the remainder distends the proximal aorta. The fraction of the SV transiently stored in the proximal aorta during the systole is subsequently released during the diastole to ensure continuous blood flow to vital organs (Fig. 5).

This elastic behaviour is described by the two-element Windkessel model, according to which the time constant of the aortic pressure decay during the diastole is the product of systemic vascular resistance and total arterial compliance. This refers to the overall compliance of the systemic arterial tree, mainly related to that of the proximal aorta. For practical reasons, total arterial compliance—a measure of this elasticity—is most often estimated using the following formula:

$$ \begin{aligned} &Total \ arterial \ compliance \ (mL/mmHg)\\&\quad=stroke \ volume/aortic \ pulse\  pressure \ [5]\end{aligned}$$

This is a calculated variable, while SV and PP are measured variables.

Because stiffness is the reciprocal of compliance, this can be expressed as:

$$ \begin{aligned}&Total \ arterial \ stiffness \ (mmHg/mL)\\&\quad=aortic\  pulse\  pressure/stroke \ volume \end{aligned}$$

From a theoretical standpoint, aortic PP is directly proportional to arterial stiffness for a given SV and to SV for a given arterial stiffness. Although these formulas overestimate “true” compliance and underestimate “true” stiffness due to the systolic run-off phenomenon, they remain useful as crude yet valid estimates. For a given MAP level, increased total arterial stiffness results from structural stiffening due to vascular wall remodeling.

### Reflection waves

An important factor which may interfere in the relationship between aortic PP, SV and aortic compliance is the presence of reflection waves. This phenomenon can be summarized as follows: arterial BP waves reflect whenever they encounter changes in arterial impedance, such as at bifurcations. The summed effect of the reflected waves generates a pressure wave travelling back to the aortic root. The aortic BP wave is therefore the combination of this backward pressure wave with the forward pressure wave produced by the left ventricular (LV) ejection.

In young individuals, the backward wave peaks at the early diastole and contributes to the diastolic aortic pressure. In cases of stiffer arteries or more proximal reflection sites due to vasoconstriction, the reflected waves return faster/earlier to the aorta, so that the aortic backward pressure wave peaks during systole. At the aortic level, this results in aortic pressure wave with a systolic peak that exceeds the maximum pressure of the forward wave. The augmentation pressure is the difference between the peak of the resulting aortic pressure and the peak of the forward pressure. As a result, in patients with stiff arteries, a first systolic peak (peak of the forward aortic pressure) and a second systolic peak (peak of the resulting aortic pressure) may be seen on the aortic pressure tracing.

The augmentation index (AIx), which is the ratio of augmented pressure to aortic PP has been considered as a measure of arterial stiffness and has been shown to be strongly associated with the occurrence of cardiovascular events and mortality [6–10]. The current pathophysiological approach commonly combines the Windkessel model with reflection phenomena. Accordingly, the increase in aortic pulse pressure (PP) associated with arterial stiffening and aging (typically after midlife) is attributed to two mechanisms: (1) proximal aortic stiffening, which increases forward pressure amplitude, and (2) wave reflection. Longitudinal studies suggest that the former mechanism may play the predominant role [11].

Additionally, arterial stiffening is associated with a slight decrease in aortic diastolic pressure. This was illustrated by large registries reporting a decrease in peripheral DAP with ageing beyond 50–55 years [11–13].

The following interpretations of aortic PP are clinically relevant:

- A high aortic PP indicates increased arterial stiffness when SV is normal or, more rarely, an abnormally high SV.
- A low aortic PP suggests a reduced SV, as abnormally decreased arterial stiffness is rare.
- A normal aortic PP reflects either a normal SV with normal aortic stiffness or reduced SV with increase arterial stiffness.

### Peripheral blood pressure

Direct monitoring of aortic BP is not feasible in practice. Carotid tonometry is a valuable non-invasive tool for capturing the aortic BP waveform, though it is not performed in current clinical practice and remains largely confined to research settings. Thus, in ICU patients, continuous monitoring of BP is feasible only with catheters placed in peripheral arteries. However, peripheral BP may differ from aortic BP so that for properly interpreting hemodynamics through peripheral BP measurements, some physiological principles should be reminded.

The relationship between aortic and peripheral PP depends on the degree of pulse wave amplification (PWA). As described above, PWA occurs as the forward aortic pressure wave, generated by the LV ejection, travels through the arterial tree, encountering progressively stiffer and narrower vessels. This alters the pressure waveform morphology, resulting in a higher PP and SAP and slightly lower DAP at peripheral sites than in the aorta, while MAP remains largely unchanged.

Several factors influence PWA and hence, the difference between aortic and peripheral PP.

- As mentioned earlier, arterial stiffening is associated with attenuated PWA. This explains why ageing is the main nonmodifiable factor associated with attenuated PWA.
- Vasoconstriction by making the reflection site more proximal also contributes to a faster return of the reflected pressure waves to the aorta and then to a decrease in the PWA phenomenon.
- Similarly, in case of short body size, the reflected pressure waves should return faster and the difference between the aortic PP and the peripheral PP should be reduced.
- During tachycardia, aortic PP decreases due to attenuation of the reflected wave, while peripheral PP remains unchanged. This results in an exaggerated difference between aortic and peripheral PP [14].
- Amongst the nonmodifiable risk factors other than age, gender is the second most important one, PWA being lower in women than in men for complex reasons.
- Traditional modifiable cardiovascular risk factors (hypertension, diabetes mellitus, hypercholesterolemia, smoking) are associated with lower PWA, while obesity has mixed effects due to the commonly associated high heart rate.

Age-related changes in arterial stiffness and PWA significantly influence the correlation between peripheral PP and SV. In younger patients, this correlation is weaker due to greater arterial compliance and more pronounced PWA. In contrast, in older patients with stiffer arteries, the correlation is stronger [15, 16].

## Clinical applications of blood pressure components

### Mean arterial pressure: the cornerstone of organ perfusion

#### Physiological insights

The MAP is an important hemodynamic parameter influenced by CO (CO = HR × SV), systemic vascular resistance, and mean right atrial pressure (RAP), although RAP underestimates the true zero-flow pressure. MAP plays a pivotal role in the autoregulation of the organs blood flow, ensuring adequate perfusion of vital organs. It is tightly regulated through homeostatic adaptations of both CO and systemic vascular resistance. In response to an acute decline in MAP, the cardiovascular system initiates compensatory mechanisms, including increased HR, increased SV (via positive inotropy, venous vasoconstriction), and systemic arterial vasoconstriction. However, these mechanisms can be impaired or overwhelmed in ICU patients, such as those with septic shock or under sedation. The relative constancy of MAP in large arteries makes it a critical variable for maintaining organ perfusion.

When MAP drops below the autoregulatory threshold, regional blood flow becomes directly proportional to MAP, increasing the risk of hypoperfusion. Accordingly, a recent meta-analysis including 34,829 ICU patients showed a significant association was found between hypotension and mortality, especially pronounced when MAP fell below 60 mmHg [17].

#### MAP targets in septic shock

Additionally, both the duration and severity of hypotension have been shown to significantly impact outcomes. For example, a retrospective cohort study of 111 patients with septic shock identified time spent below a MAP of 65 mmHg and the depth of hypotension as independent predictors of 30-day mortality [18]. More recently, in a nationwide cohort study, Khanna et al. reported that MAP values < 65 mmHg were linked to worse outcomes, with the lowest risk observed at ~ 65 mmHg in general ICU, ~ 70 mmHg in sepsis, and ~ 72 mmHg in septic shock [19]. Although there is no universally accepted MAP threshold to guarantee pressure-independent perfusion across all organs, current guidelines for septic shock recommend achieving a MAP of at least 65 mmHg to prevent organ hypoperfusion [20].

#### MAP targets in hypertensive patients

**Fig. 6:**
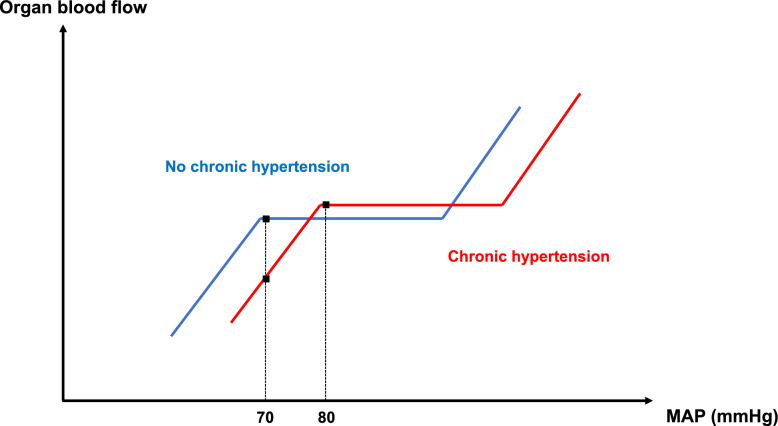
Autoregulation curves of organ blood flow based on the presence (red) or absence (blue) of chronic hypertension

In patients with chronic hypertension, higher MAP targets may be necessary. This suggestion is based on the rightward shift of the autoregulation curve for organ blood flow observed in hypertensive individuals (Fig. 6). In non-hypertensive patients, a MAP of 70 mmHg should typically fall within the plateau phase of the autoregulation curve, ensuring stable blood flow. In contrast, in hypertensive patients, the same MAP may lie within the descending portion of the curve, leading to reduced regional blood flow and an increased risk of hypoperfusion (Fig. 6).

A retrospective analysis of a large database compared outcomes in three subgroups of septic patients with a history of chronic hypertension according to their average MAP at ICU admission [21]. The group with an average MAP ≥ 80 mmHg experienced fewer episodes of acute kidney injury (AKI) compared to those with MAPs between 73 and 80 mmHg, and those with MAP < 73 mmHg [21]. Notably, 30-day mortality was lower in the 73–80 mmHg MAP subgroup compared to the two other subgroups [21]. However, it is important to note that the proportion of patients with septic shock was low, even in the subgroup of MAP < 73 mmHg [21]. A randomized controlled trial (RCT) by Asfar et al. [22] evaluated the impact of higher MAP targets in patients with septic shock. The study compared a MAP target of 80–85 mmHg with a target of 65–70 mmHg and found that the higher target improved renal function and reduced the need for renal replacement therapy in patients with a history of chronic hypertension [22]. However, no significant differences were observed in 28-day or 90-day mortality [22]. In contrast, these findings were not replicated in a more recent RCT conducted in Japan by Endo et al. [23]. This multicentre, pragmatic, open-label trial enrolled patients aged ≥ 65 years with septic shock and compared MAP targets of 65–70 mmHg versus 80–85 mmHg. Surprisingly, the group assigned to the higher MAP target experienced significantly increased mortality [23]. Among patients with prior chronic hypertension, there were no differences in mortality or renal function between the two MAP target groups [23]. A key aspect of the Endo et al. study was the early initiation of vasopressin—as soon as a norepinephrine dose of ≥ 0.1 μg/kg/min was required to achieve the target MAP. This led to a threefold higher cumulative vasopressin dose in addition to increased norepinephrine exposure in the high-MAP group [23]. While this unconventional strategy might have contributed to the observed excess mortality [24], it is not possible to determine whether this effect was related to vasopressin, norepinephrine, their combination, or other unmeasured factors. More likely, the need for higher MAP targets in non-hypertensive patients resulted in greater overall vasopressor exposure, which may explain the signal for harm [23]. By contrast, in a prior RCT of patients aged ≥ 65 years [25], where median MAPs were 72.6 mmHg in the high target group and 66.7 mmHg in the low target group, no difference in mortality was observed. Notably, vasopressin was rarely used in this study. Similarly, in the Asfar et al. trial, vasopressin was not used at all [22].

In summary, although definitive conclusions about the optimal MAP target in patients with chronic hypertension remain elusive, a pragmatic approach can be proposed based on physiological reasoning and available clinical data: If shock persists despite achieving a MAP of 65–70 mmHg, targeting a higher MAP may be considered [26, 27]. In this regard, a short “vasopressor test,” consisting of a transient increase in MAP (≈30 min), has been proposed to assess peripheral perfusion, thereby identifying patients who may benefit from higher MAP targets [28]; this individualized approach was applied in large RCTs such as ANDROMEDA-SHOCK [29] and ANDROMEDA-SHOCK-2 [30].

#### MAP targets in patients with elevated central venous pressure

In patients with elevated central venous pressure (CVP), managing MAP requires careful adjustment to ensure adequate organ perfusion [31]. CVP acts as the downstream pressure opposing perfusion of critical organs, such as the brain and kidneys. The mean perfusion pressure (MPP) for these organs is calculated by the difference between MAP and CVP (MPP = MAP—CVP). While MAP alone is often used as a surrogate for MPP in cases of low CVP, this approximation becomes unreliable when CVP is elevated. An increase in CVP reduces MPP, which can impair organ perfusion and increase the risk of hypoperfusion. This highlights the importance of incorporating CVP into the assessment of perfusion adequacy [31]. Indeed, Ostermann et al. demonstrated that MPP—but not MAP alone—is an independent predictor of AKI progression, identifying a critical MPP threshold of 60 mmHg [32]. Evidence from both cardiac surgery and heart failure populations also suggests that MPP is a critical but often overlooked determinant of organ perfusion [33, 34]. Lower MPP has been associated with a higher risk and persistence of acute kidney injury after cardiac surgery, while in heart failure it reflects the interplay between arterial pressure and venous congestion, underscoring its potential value as a bedside hemodynamic target [33, 34]. When elevated CVP compromises MPP, the primary management goal should be to reduce CVP whenever possible since lowering CVP also reduces the risk of venous congestion [35], a known contributor to organ dysfunction, especially AKI [36]. If immediate CVP reduction is not feasible, increasing MAP may be considered as a secondary strategy to restore sufficient MPP and prevent hypoperfusion; however, this approach has not yet been validated and should be tested in future clinical studies. Of note, in abdominal compartment syndrome, elevated intra-abdominal pressure may reduce kidney perfusion pressure by raising downstream venous pressure and hence, may contribute to kidney injury even when MAP is apparently preserved.

### Pulse pressure: a surrogate for stroke volume

#### Physiological insights

As we mentioned above, SV and arterial stiffness are the main determinants of aortic PP [37, 38]. We also earlier detailed the relationships between peripheral PP and aortic PP. In clinical practice, for a given SV, peripheral PP is higher in older than younger individuals since arterial stiffness is more marked in the former [12]. As an example, a PP of 40 mmHg in older patients could be a good marker of a low SV, while its interpretation is less straightforward in younger patients. In the absence of CO monitoring, an important clinical question is whether changes in peripheral PP can reflect changes in SV during diagnostic tests such as passive leg raising (PLR) or during therapeutic interventions such as fluid administration or norepinephrine infusion.

#### Clinical implications

The results of clinical studies addressing this question are variable. Some studies showed a good correlation [15, 16, 39], while others were less positive [16, 40]. As mentioned above, age through arterial stiffness plays an important role. Accordingly, a better correlation between fluid-induced changes in CO and in peripheral PP was shown in elderly patients than in younger ones [15, 16]. The severity of sepsis [40, 41] and the administration of norepinephrine [16] are potential factors leading to a decoupling of these variables. Nevertheless, recent studies showed that the changes in peripheral PP—as surrogates of changes in SV—during dynamic tests such as PLR [42], decrease in positive end-expiratory pressure [43], sigh manoeuvre [44] and end-expiratory occlusion [45] can be used to predict fluid responsiveness with acceptable accuracy in ICU patients. Recently, a PP of less than 40 mmHg was used to define a low SV in the intervention group of ANDROMEDA-SHOCK-2, a multicentre RCT in patients with septic shock [30]. The intervention group, which relied on capillary refill time, PP, DAP, and fluid responsiveness indices as the main variables guiding therapeutic interventions, was compared to standard care in a total of 1,500 patients [30]. Additionally, the ongoing ANDROMEDA-PEGASUS study (NCT06737614) addressing the relationship between PP and SV is expected to deliver large-scale epidemiological data across different ICU contexts.

### Diastolic arterial pressure: a marker of vascular tone

#### Physiological insights

The DAP is a marker of vasomotor tone. The following arguments, emphasize the importance of DAP in diagnosis and therapeutic decision-making in shock states.

**Fig. 7:**
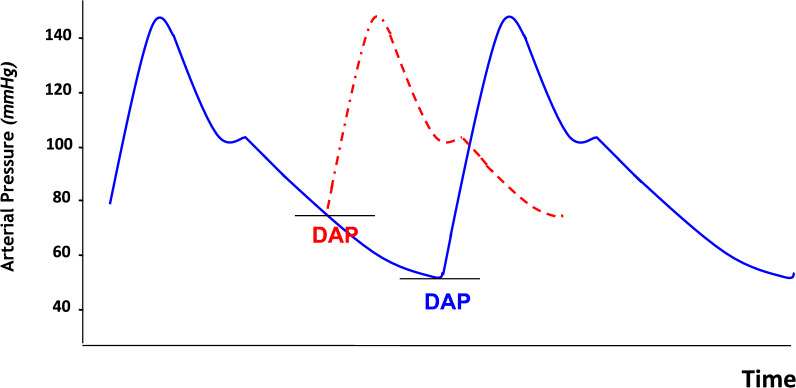
Effect of heart rate on diastolic arterial pressure (DAP). An increase in heart rate reduces the diastolic time and results in higher DAP

Low DAP primarily reflects depressed arterial tone, which is a hallmark of septic shock due to vasodilation and reduced vasomotor responsiveness. A previous study [37] showed a close relationship between DAP and peripheral resistance highlighting its value as a marker of vascular tone. As such DAP may help guide the decision to initiate norepinephrine. Nevertheless, it is essential to interpret DAP according to HR. Indeed, at high heart rates, DAP may be higher than normal due to the reduced diastole duration (Fig. 7), even if the vascular tone is normal. In this regard, a DAP of 50 mmHg in a patient with a HR of 90 bpm suggests higher vasomotor tone than in a patient with a HR of 150 bpm and the same value of DAP [46].

Arterial stiffness is another important determinant of DAP [37] as illustrated by large registries that reported a decline in DAP as age increases above 60 years [13, 47]. Notably, the low DAP due to arterial stiffening [13, 47] never reaches values as low as those encountered in vasodilatory shock [16].

Additionally, DAP is critical for the left coronary perfusion, serving as the upstream pressure for LV blood flow during diastole. Therefore, a persistently low DAP increases the risk of myocardial ischemia, particularly in patients with prior coronary artery disease.

Finally, DAP remains quasi-constant from the aorta to the periphery, while peripheral SAP and PP increase due to the PWA phenomenon, ensuring its value as a bedside clinical indicator.

#### Clinical implications

As mentioned above, DAP as a marker of vascular tone can help guide the initiation of norepinephrine in shock states [46, 48]. Observational studies have demonstrated that low DAP values are associated with adverse outcomes, including increased mortality, AKI, and myocardial injury [49, 50]. In a retrospective observational study including 77,328 septic patients, a DAP threshold of 48 mmHg (and 44 mmHg in patients with septic shock) was identified, below which the likelihood of AKI, myocardial damage and mortality rose significantly [19]. This association underscores the importance of DAP in guiding therapeutic interventions, such as norepinephrine administration, to restore vascular tone and improve perfusion.

#### New perspectives

Recently, the diastolic shock index (DSI), defined as the ratio HR to DAP was proposed as more robust marker of vascular tone than DAP [51]. A clinical study evaluated DSI at pre- norepinephrine and norepinephrine initiation points and showed a strong association of higher DSI values with increased mortality [51]. Importantly, neither HR nor DAP alone reliably predicted outcomes. The predictive performance of DSI was comparable to established markers like SOFA score and lactate, outperforming MAP and “systolic” shock index [51]. Early initiation of norepinephrine was found to benefit to patients with elevated DSI, highlighting its potential to guide timely interventions [51]. The DSI thus may emerges as a critical tool for identifying high-risk septic shock patients and optimizing resuscitation strategies.

Finally, the ratio of DAP to the product of HR and the NE dose in µg/kg/min (DAP/ (HR x norepinephrine dose)) was proposed as an index of the vasomotor tone responsiveness to NE (VNERi) in patients with septic shock evaluated at the time of initiation of NE [52]. In a post-hoc analysis of the ANDROMEDA-SHOCK database, VNERi demonstrated the strongest association with in-hospital mortality compared to DAP, DAP/HR and MAP/NE dose, emerging as the most significant covariate in a multivariate model [52]. The model revealed an inverted J-shaped relationship between in-hospital mortality and VNERi, with a nadir point at 6.7, below which mortality increased [52]. Further studies are required to investigate whether VNERi could be integrated into decision-making of early septic shock, especially for the decision to add another vasopressor to NE [52]. When VNERi suggests poor responsiveness to norepinephrine, adding a vasopressor with a different mechanism of action (e.g. vasopressin) might be physiologically sound, rather than simply escalating catecholamine dose. This approach minimizes catecholamine load and targets complementary pathways of vascular tone regulation.

In summary, DSI may emerge as a critical tool for identifying high-risk septic shock patients and optimizing resuscitation strategies, while the VNERi provides complementary information on vascular responsiveness to norepinephrine; together, these parameters could serve as personalized inclusion criteria for future trials, for instance by enrolling patients with vasoplegia and/or vascular unresponsiveness to norepinephrine defined by high DSI and low VNERi rather than solely low MAP. Furthermore, in the perioperative setting, VNERi could help distinguish true vascular hyporesponsiveness from pharmacologically-induced vasoplegia, although this hypothesis still requires validation in dedicated cohorts.

### Systolic arterial pressure: a marker of left ventricular afterload

#### Physiological insights

Aortic SAP is a main determinant of LV afterload, contributing to myocardial wall stress during systolic ejection [53]. According to Laplace's law, wall tension is directly related to pressure and ventricular radius while inversely proportional to wall thickness: *T* = P * r / 2 h, where T is wall tension (stress), P is LV end-systolic pressure, r is LV radius, h is LV wall thickness.

LV end-systolic pressure can be estimated using the formula: LV end-systolic pressure = 0.9 × aortic SAP. In the absence of aortic stenosis or outflow tract obstruction, the higher the aortic SAP, the greater the force the left ventricle must exert to open the aortic valve and eject blood into systemic circulation (Fig. 5).

As detailed above, peripheral SAP can better reflect aortic SAP and hence, LV afterload in patients with stiff arteries compared with young and healthy individuals for whom PWA is more pronounced [54, 55]. In other words, a peripheral SAP of 140 mmHg indicates a higher LV afterload in older patients than in younger subjects. At the other end of the spectrum, Khanna et al. recently demonstrated in a large cohort of septic ICU patients that low SAP values (< 100–110 mmHg) were strongly associated with increased mortality [19].

#### Estimation of aortic systolic arterial pressure

Recently, based on physiological grounds, Chemla et al. proposed a new empirical formula for estimating aortic SAP non-invasively: the MAP^2^/DAP ratio [56]. The formula was tested across three datasets: the first of 139 patients with high-fidelity aortic pressure measurements, the second including 64 patients with simultaneous aortic and brachial artery measurements, and the third was a cohort of 30 ICU patients with radial artery catheters [56]. The analysis of the three datasets, showed that aortic SAP can be reliably estimated from the MAP^2^/DAP ratio, provided measurement errors are minimized, suggesting potential applications for cardiovascular risk assessment in large BP databases [56]. Further studies are required to investigate whether the changes in the difference between (MAP^2^/DAP) and DAP (surrogate of aortic PP) would perform better than peripheral PP to assess the changes in SV during diagnostic or therapeutic interventions.

## Pulse pressure variation: a dynamic marker of fluid responsiveness

### Physiological insights

**Fig. 8:**
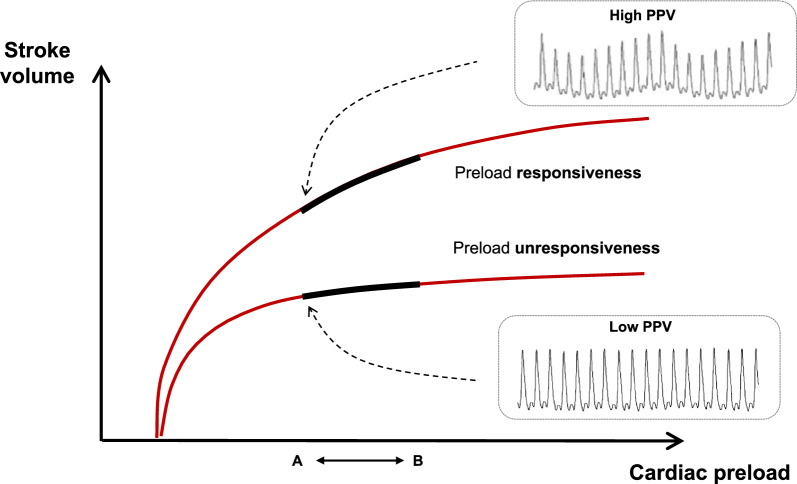
Pulse pression variation (PPV) and Frank-Starling mechanism. Significant increase in stroke volume (SV), which defines preload-responsiveness (blue) is associated with a high PPV. Small increase in SV, which defines preload-unresponsiveness (red), is associated with a low PPV

Fluid responsiveness is generally defined as the ability of the heart to significantly increase its SV or CO in response to a fluid bolus [57]. Physiologically, this implies that both ventricles are preload-responsive, meaning that they operate on the ascending portion of the curve describing the relationship between SV and preload (Frank-Starling mechanism) (Fig. 8).

During mechanical ventilation, cyclic changes in SV should occur also in cases of biventricular preload responsiveness, SV being minimal during the expiratory phase [57–60]. Consequently, it has been hypothesized that the magnitude the respiratory changes in SV should reflect the magnitude of fluid responsiveness [58, 59]. Aortic PP depends on both SV and arterial stiffness, and aortic stiffness is expected to remain essentially unchanged over the respiratory cycle, as it mainly depends on structural arterial properties which remain constant over short-term. As a result, the respiratory variation of aortic PP (and of peripheral PP) should reflect the respiratory variation of SV [58–60]. Therefore, the respiratory variation of PP, called PP variation (PPV) should be a marker of fluid responsiveness in patients who receive mechanical ventilation [58–60].

### Clinical implications

Since the publication of the first study that showed that PPV remarkably predicts fluid responsiveness in patients with septic shock ventilated with a tidal volume of at least 8 mL/kg [61], numerous studies in different settings have confirmed the reliability of this dynamic predictor of fluid responsiveness [62].

Initially calculated manually, PPV is now measured automatically by conventional hemodynamic monitors, which provide real-time values for clinical use. In more advanced monitors, algorithms to estimate SV based on BP wave analysis have been implemented, enabling real-time CO monitoring [63].

The three main limitations of using PPV to predict fluid responsiveness during mechanical ventilation are (1) the presence of cardiac arrhythmias, (2) the use of tidal volume lower than 8 mL/kg and [64, 65], (3) the presence of spontaneous breathing activity [42, 66]. In these situations, other fluid responsiveness indices have been proposed [67], most of these indices requiring measurements of CO. Nevertheless, it has been proposed in some situations to measure the changes of PPV during dynamic tests [67]. This approach has the advantage not to require any CO monitor.

In patients mechanically ventilated with a tidal volume of 6 mL/kg, it has been proposed to perform a tidal volume challenge (TVC), which consists of measuring the change of PPV during a 1-min increase in tidal volume from 6 to 8 mL/kg [65]. This method was shown to be performant to predict fluid responsiveness in various settings [65, 68], including mechanical ventilation during prone position [69] and to a lesser degree during mechanical ventilation with persistent spontaneous breathing activity [42]. A difference between PPV measured at 8 mL/kg and PPV measured at 6 mL/kg higher than 3.5% was generally reported as a cut-off value above which fluid responsiveness is present in supine [65] and in prone position [69].

Additionally, recent studies have suggested to measure the changes of PPV during PLR as a reliable alternative for assessing fluid responsiveness in patients ventilated with low tidal volume [70, 71]. A reduction in PPV during PLR has demonstrated strong predictive accuracy in this setting [70, 71]. Like TVC, this method does not require any CO monitoring device to be performed. Hamzaoui et al. tested the predictive value of the changes of PPV during PLR in mechanically ventilated patients with persistent spontaneous breathing activity [42]. They showed that the decrease in PPV during PLR better predicted fluid responsiveness than the absolute PPV values in this population [42]. Although the predictive capacity of this method in this setting [42] was not as perfect as that reported in studies including patients with no inspiratory efforts [70, 71], its good sensitivity makes it valuable for predicting fluid responsiveness in mechanically ventilated patients with inspiratory efforts for whom CO monitoring is often not used.

## Conclusions

**Fig. 9:**
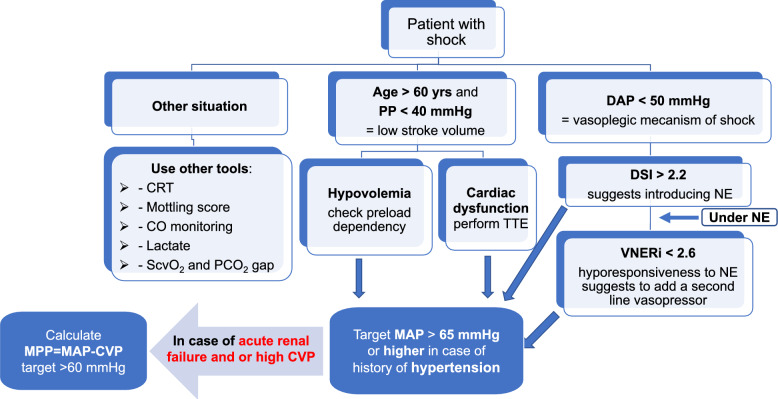
A simplified algorithm illustrating how arterial pressure components may assist clinical decision-making in patients with shock. It is important to note that the algorithm does not encompass all possible clinical scenarios; rather, it highlights selected situations where specific components of arterial pressure may be particularly informative. CO: cardiac output; CRT: capillary refill time; CVP: central venous pressure; DAP: diastolic arterial pressure; DSI: diastolic shock index; MAP: mean arterial pressure; MPP: mean perfusion pressure; NE: norepinephrine; PCO_2_ gap: difference in carbon dioxide pressure between the central venous blood and the arterial blood; ScvO_2_: central venous oxygen saturation; TTE: transthoracic echocardiography

Analysis of the components of invasive BP blood pressure (SAP, DAP, MAP, PP) in critical care settings provides essential hemodynamic information that help optimize therapeutic interventions (Fig. 9). For accurate interpretation, it is crucial to rigorously assess the quality of the arterial pressure waveform to avoid measurement artifacts such as under- or over-damping. MAP, which remains constant throughout the arterial tree, serves as a therapeutic target but should be adjusted based on the patient’s medical history (particularly chronic hypertension) and CVP values. DAP, an indicator of vasomotor tone, varies minimally from the aorta to peripheral arteries and can serve as a reliable trigger for initiating norepinephrine therapy. In addition, the DSI may, in certain clinical contexts, be more informative than DAP alone in guiding the initiation or escalation of norepinephrine therapy. Moreover, the newly proposed VNERi ratio (DAP/[HR × norepinephrine dose]) may help identify patients with a poor response to norepinephrine and could serve as a trigger for the early initiation of a second vasopressor, such as vasopressin. SAP and PP typically increase from the aorta to peripheral arteries, with the magnitude of this increase primarily depending on age. In older patients with stiff arteries, peripheral SAP and PP (and their changes) can reflect LV afterload and SV (and their changes), respectively. PPV and its changes during TVC and PLR are valuable tools for assessing fluid responsiveness in patients receiving mechanical ventilation.

## Data Availability

Not applicable.

## Abbreviations

BP: Blood pressure
SAP: Systolic arterial pressure
DAP: Diastolic arterial pressure
MAP: Mean arterial pressure
PP: Pulse pressure
DSI: Diastolic shock index
VNERi: Vasomotor tone responsiveness to NE index
HR: Heart rate
SpO2: Peripheral oxygen saturation
CO: Cardiac output
PPA: Pulse pressure amplification
SV: Stroke volume
PWA: Pulse wave amplification
LV: Left ventricular
RAP: Right atrial pressure
ICU: Intensive care unit
AKI: Acute kidney injury
NE: Norepinephrine
SSC: Surviving sepsis campaign
CVP: Central venous pressure
MPP: Mean perfusion pressure
PLR: Passive leg raising
RCT: Randomized controlled trial
PPV: Pulse pressure variation
TVC: Tidal volume challenge
